# Rapid and cyclic dust accumulation during MIS 2 in Central Asia inferred from loess OSL dating and grain-size analysis

**DOI:** 10.1038/srep32365

**Published:** 2016-09-02

**Authors:** Yun Li, Yougui Song, Zhongping Lai, Li Han, Zhisheng An

**Affiliations:** 1 State Key Laboratory of Loess and Quaternary Geology, Institute of Earth Environment, Chinese Academy of Sciences, 97 Yanxiang Road, Xi’An 710061, China; 2 The Salt Lake Geology and Environment Laboratory, Qinghai Institute of Salt Lakes, Chinese Academy of Sciences, 18 Xining Road, Xinning 810008, China; 3 School of Earth Sciences, China University of Geosciences, Wuhan 430074, China; 4 Salt Lake Analytical and Test Department, Qinghai Institute of Salt Lakes, Chinese Academy of Sciences, 18 Xinning Road, Xining 810008, China

## Abstract

Due to lack of reliable proxies from the Westerlies-dominant region, the strength change of Northern Hemisphere Westerlies remains poorly understood. The aim of this study is to provide a reliable paleoclimatic proxy about the Northern Hemisphere Westerlies change. Here we report a 30.7 m thick loess section from the Ili basin directly controlled by the Westerlies. Based on optically stimulated luminescence (OSL) and high resolution grain-size records, we reconstruct the change history of the Westerlies strength during the last glacial period (mainly Marine Isotope Stages 2, MIS2), being similar with the Westerlies index recorded in the Qinghai Lake sediments. Within error limits, all ages are in stratigraphic order. We further compare the climatic records among the Ili loess, Qinghai Lake and the NGRIP, their similarity shows a good climatic coupling relationship among the Central Asia, East Asia and the North Atlantic, and the Westerlies plays a critical influence in transporting the North Atlantic signal to Central and East Asia.

Aeolian dust may play an important role in climate forcing by altering the radiation balance in the atmosphere through direct effects on radiation at both solar (shortwave) and terrestrial (longwave) portions of the electromagnetic spectrum^1^,^2^. Fine-grained dust can be a significant carrier of Fe and phytoplankton blooms can occur in the ocean after dust-derived Fe fertilization^3^. Such blooms can result in significant carbon dioxide drawdown from the atmosphere, thus changing carbon balance between ocean and atmosphere. Loess, a geologic record of dust, is aeolian sediment dominated by silt-sized particles. So studying the loess record may provide insight into past atmospheric circulation patterns and paleoclimatic changes.

The Asian continent is a major source of aeolian dust, not only deposited on the North Pacific^4^ but also deposited on the Greenland Ice Core^5^. Loess deposited in the Asian continent can be regarded as one of the classical loess regions in Earth, including the Central Asia loess and loess in the Chinese Loess Plateau (CLP). However, in contrast to the widely investigated loess deposits in the CLP, Central Asian loess–paleosol sequences are still insufficiently known and poorly understood. During the Quaternary period, accumulation of wind-blown dust formed a widespread and thick loess cover at the piedmonts of the mountains in the Central Asia, and can be used to reconstruct the atmospheric circulation patterns controlled by the Westerlies^6^,^7^.

Grain-size variations of loess have been widely used as a proxy of past variations in both aeolian dynamics and patterns of atmospheric development^8^,^9^. And, on millennial timescales, evidence for rapid grain-size variations in the worldwide loess has been interpreted as a possible consequence of Heinrich ice-rafted events and the Dansgaard/Oeschger (D-O) events in the Atlantic^9^,^10^,^11^,^12^. This climatic teleconnections has been interpreted as the transportation of the Westerlies linking the North Atlantic and the Eurasia loess^9^,^10^,^13^. However, this climatic coupling relationship between the North Atlantic and East Asia is mainly based on climate simulations^9^, it has a noticeable lack of the Westerlies intensity proxy in the Westerlies-controlled Central Asia.

The Ili Basin, an intermontane basin defined by west-facing trumpet shaped mountains, is climatically controlled by the Westerlies (Fig. 1), which carries adequate moisture from the Atlantic Ocean, Mediterranean Sea, Black Sea and Caspian Sea^14^. Mean annual precipitation in the basin is the largest in Xinjiang, which is 200–400 mm on the plains, and can reach 800 mm in the mountain zones^15^. Mean annual temperature in the Ili basin varies from 2.6 to 9.2 °C depending on the terrain.

Here, we report 15 optically stimulated luminescence (OSL) ages for a loess section in the Ili basin on the eastern Central Asia, and use high resolution grain size record of loess to calculate the Westerlies intensity change during MIS 2. It shows a striking match among temperature variations over Greenland, the grain-size change of the Ili loess and the Westerlies index (WI) from Lake Qinghai (QHH) sediments, their similarity showed the northern Westerlies passed the climatic change from the North Atlantic through Central Asia to East Asia during MIS 2.

## Results

The Xiaoerbulake (XEBLK) section (83.07°E, 43.42°N, 1050 m above sea level) was located on the seventh terrace of the Kunes River, a branch of Ili River in the east Ili Basin. The loess section can be divided into seven layers which include three loess layers, one paleosol layer and two weak developed soil layers and one modern soil layer. Lithological descriptions of the XEBLK section are presented in Table S1.

Fifteen OSL ages (Table 1) gradually increases from 0.46 to 29.04 ka with increasing depth, and there are no obvious dust accumulation breaks at the current luminescence sampling resolution. The association of the grain-size analysis and the OSL dates clearly indicates a highly variable deposition rate. This restricts the usefulness of the normally used simple interpolation techniques for constructing an age model. Two OSL date at 24 and 26 m are significantly reversed even when taking into account the dating errors. Independent OSL age models can be generated by linear interpolation of OSL ages after excluding the two abnormal OSL age.

Here, we calculate the mean mass accumulation rate (MAR) at XEBLK using the linear fitting of depth with OSL ages, which was also adopted for the CLP loess. The MIS 2 stadial (2–26 m) of the XEBLK section is equivalent to age of the L_1-1_ loess in CLP, and its mean MAR (141 cm/ka) is higher than those (7.0 cm/ka) of the Weinan section in the south CLP^16^, (80 cm/ka) of the Jingyuan section in the central CLP^17^, and (98 cm/ka) of the Yuanbao section in the western CLP^18^, but similar to (152 cm/ka) of TLD^19^ in the Ili basin. An intriguing similarity is noted between the dust concentrations of Guliya ice core and the MAR of XEBLK section during 11 and 29 ka (Fig. S1), which supports the validity of the OSL chronology.

The XEBLK Loess samples indicate grain size variation between four consecutive classes ranging from clay (<4.6 μm), fine silt (4.6–20.7 μm), coarse silt (20.7–63.4 μm) and fine sand (63.4–153.8 μm). Five main zones can be distinguished, which can be interpreted as corresponding to different climate regimes (Fig. S2). Above the 2.5 m depth (Zone 1) the clay content regularly increases, from 6% to 7%, while the coarse silt percentage deceases from 43% to 40%. The fine silt content fist decreases to 30%, then returns to values of 40%, similar to the fine sand content changes. Between 2.5 and 9 m depth (Zone 2), the coarse silt and fine sand fractions gradually increases, while the clay and fine silt content show this pattern inversely. This increase in coarse silt and fine sand may be interpreted as being related to a strengthening in the wind regime. From 12 m to 9 m depth (Zone 3), the proportions of the different grain size classes changes abruptly, with the coarse silt and fine sand decreasing, while the clays and fine silt and clay content increase evidently. Considering all the material as windblown, the increasing fraction of finer material might be interpreted as reflecting an abrupt weakening of the wind dynamics. In the interval between 24.5 m and 12 m (Zone 4), the proportion of fine grained material decreases while coarse loam shows an opposite trend. Such pattern seems to show a strengthening in the wind dynamics responsible for the dust transportation. The last interval, between 2 m and the bottom of the sequence, indicates a decrease in the proportion of coarse particle contrary to an increase in fine material suggesting a decrease in the wind regime (Zone 5).

## Discussions

**Figure 2:**
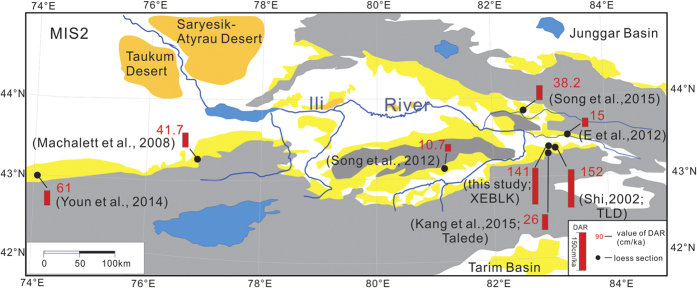
The mean MAR of the loess from the Ili basin during MIS 2. The map was plotted using CorelDRAW X7 (http://www.corel.com/cn/).

We have complied information from eight loess sections^20^,^21^,^22^,^23^, for which detailed and reliable chronological information is available (Fig. 2). The mean MAR of the Ili loess during MIS2 varied greatly from 10.7 to 152 cm/ka along the Tianshan, but we did not observe a clear west-east or a north-south pattern in MAR of the Ili loess for MIS 2 along the Tianshan. So we consider that the difference of the MAR mostly reflect the strong influence of local topography and the distance between dust source and loess site. For example, the MAR of our section during the MIS 2 is similar with the TLD section (Fig. 2)^19^. However, the MAR of the Talede^23^ is obviously lower than those of our section (Fig. 2), maybe because its location in the highest terraces of the Ili River, the poor-developed river terrace provides an unsteady sedimentary environment and leads to a smaller deposition rate than our section and the TLD section (Fig. S3)^19^,^24^.

**Figure 3:**
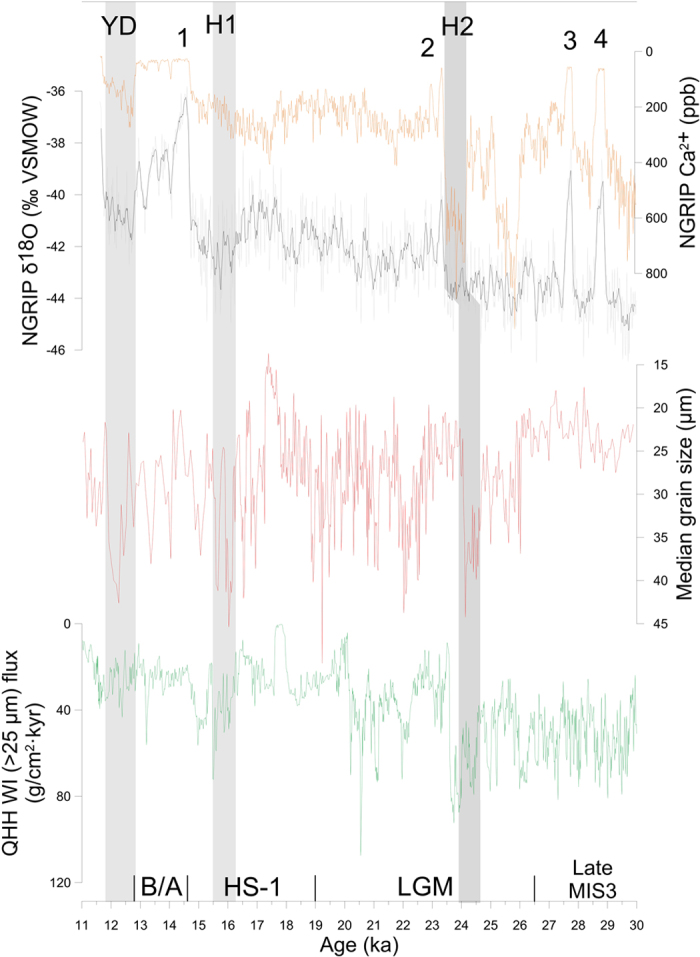
Comparison of Central/East Asia and North Atlantic climate records. XEBLK (red) median grain size with NGRIP δ^18^O (black, three-point running mean) and Ca^2+^ (orange) records^5^,^11^,^38^ and Qinghai Lake WI (green)^28^. Gray bars denote the Heinrich-like events identified in the three records. Black numbers (1–4) denote well aligned DO events identified in the three records.

Coarsening of median grain size around the ages of 12, 16 and 24 ka indicates that the North Atlantic Heinrich events (YD, H_1_ and H_2_) are associated with strong Westerlies circulation in the Central Asia (Fig. 3). D-O 1–4 are characterized by fining of median grain size in the Central Asia, and well aligned between the Qinghai Lake grain-size and ice-core records. Moreover, the Central Asia loess and the Qinghai Lake show greater variation in grain size in response to slight change of the Greenland temperature, especially for grain-size decreasing event at ~17.5 ka (Fig. 3), matching a significant improvement of deep-water ventilation indicated by increasing whole shell at ~17.5 ka^25^ and decreasing ^231^Pa/^230^Th data at ~19 ka^26^ in the North Atlantic. The pattern of the grain-size changes of the XEBLK section is similar to WI of the Qinghai Lake sediments in the western China (Fig. 3). For example, from 11 ka to 16 ka and 18 ka to 22 ka, the grain size of the XEBLK and the Qinghai Lake gradually became coarser, and between 24 and 26 ka they simultaneously decrease. Moreover, grain-size fining of the XEBLK section between 26 and 30 ka is accorded with the warm and wet climate during MIS3 27. The validity of the OSL chronology is also supported by the good correlation between grain-size change of XEBLK and WI of Qinghai Lake. The correlative nature of these DO and Heinrich events in the ice-core, loess in the Central Asia and lake in the western China directly links millennium-scale variability in the Central Asia to that in Greenland and East Asia during MIS2. However, grain size change at XEBLK is opposite for that at QHH before 26 ka BP. An *et al*.^28^ suggests, during 24–30 ka, the QHH sedimentary facies is loess-like silt and fine sand layers, maybe during the warmer and wetter MIS 3, the increasing precipitation bring a little of fine sand into the lake and increases the grain size. But during the late MIS 3, the decreasing Westerlies let to the fining grain size of XEBLK loess. And the NGRIP δ^18^O and Ca^2 +^ records and Qinghai Lake WI also show wetter and warmer climate from 30 ka to 11 ka, but the grain size of Ili loess shows an opposite trend. we find, on millennial timescales and long trend, the grain size of the Stayky loess from the eastern Europe^29^ is similar with that of XEBLK, showing H1 is colder than LGM, wetter and warmer climate from 30 to 16 ka and colder and dryer climate form 16 to 11 ka (Fig. S4). This similarity shows the westerlies transport the climatic signal of the Europe to the Central Asia.

Sun *et al*.^9^ used the water-hosing experiments to simulate that the slow-down of Atlantic meridional overturning leads to the strengthening of the Westerlies during LGM. The fact that XEBLK locating at typically Westerlies-dominant region and the similarity of grain size between XEBLK loess in the eastern Central Asia and Qinghai Lake in the East Asia, supports our interpretation that the Westerlies play an critical influence in transporting the North Atlantic signal to the East Asia. Moreover, recent global climate modeling results suggested that the increased meridional SST gradient in the North Atlantic at the LGM, associated with an increase in the polar sea ice, not only shifts the Westerlies equatorward, but also strengthens the Westerlies^9^,^30^,^31^,^32^. At the same time, during the LGM, the Westerlies strength displays an abrupt change towards a stronger and southward shift of the storm track^31^,^33^. In a word, these climate simulation and geological records further support the strengthening of Westerlies transport the climatic signal in the North Atlantic to the Central Asia and East Asia during MIS 2.

## Conclusions

The chronology of XEBLK profile in the Ili Basin was established using OSL dating, ranging from 0.46 to 29.04 ka. Very high MAR of 141 cm/ka was observed during MIS 2. The variation of the MAR in the basin is interpreted to due to the local factors, strong influence of topography and the distance between dust source and loess site. Using grain-size data, we reconstruct changes in the strength of the Westerlies during the 11 ka and 30 ka and find reconstructed millennial-scale variations that are broadly correlated with temperature variations over Greenland, and WI of Qinghai Lake sediments, suggesting that the northern Westerlies play a role in transmitting the signal from the North Atlantic to the Central/East Asia.

## Methods

### OSL dating

A total of 15 OSL samples were collected from the XEBLK section by hammering aluminum tubes (20 cm long cylinder with a diameter of 6 cm) into freshly cleaned vertical sections (Table 1). In laboratory, samples at each end of the tube were scraped away and used for dose rate measurement, and the unexposed materials in the middle part of the tube were used for equivalent dose (*D*_e_) estimation.

Sample preparation was similar to the procedures by Lai and Wintle^34^. Quartz fraction of 38–63 μm was extracted after a series of treatments using HCl, H_2_O_2_, and fluorosilicic acid; its purity was checked by infrared stimulation, and no obvious infrared stimulated luminescence was observed from any sample. Quartz grains were then mounted on stainless steel discs (9–10 mm in diameter) with silicone oil.

Luminescence measurements were performed using an automated Risø TL/OSL DA-20 reader. The OSL signal was detected by a 9235QA photomultiplier tube through a 7.5 mm Hoya U-340 filter. Irradiation was carried out using a ^90^Sr/^90^Y beta source. OSL stimulation was carried out for 40 s at 130 °C. OSL signals from the initial 0.64 s of stimulation were integrated for growth curve construction after background subtraction. The stimulation used blue diodes (λ = 470 ± 20 nm) for quartz OSL, and infrared diodes (λ = 830 ± 10 nm) for feldspar infra-red stimulated luminescence (IRSL). A á value of 0.035 ± 0.003 for quartz^35^, and 0.1 ± 0.01 for feldspars was used. The single aliquot regenerative-dose (SAR) protocol was used for *De* determination^36^.

Lithogenic radionuclide activity concentrations were determined from measurements of U, Th and K concentrations using neutron activation analysis (NAA) of dried and ground bulk samples. The cosmic ray dose was estimated for each sample as a function of depth, altitude and geomagnetic latitude^37^.

The concentrations of Uranium (U), thorium (Th) and potassium (K), the water content and the calculated dose rate are listed in Table 1. U, Th and K contents range from 4.984 to 2.797 ppm, 12.92 to 9.809 ppm, 1.479 to 2.197%, respectively, resulting in a dose rate between 2.84 to 4.09 Gy/ka. The *De* values increase with depth from 1.68 to 87.89 Gy. Our OSL ages are generally in stratigraphic order with depth (Fig. S5).

For *De* determination, 12–16 aliquots were measured for each sample to calculate a final *De*. Fig. S6A, B show growth and decay curves for XEBLK-12. The OSL signal decreases very quickly during the first second stimulation (Fig. S6A), which indicates the OSL signal is ‘fast component’ dominant. Recuperation was in all cases negligible (<3%), and for most of the aliquots the recycling ratios fall into the range of 0.9–1.1. A few discs with a recycling ratio falling outside this range were rejected in the final *De* calculation. The growth curve was fitted by linear plus exponential function.

### Grain size analyses and interpretation

Grain-size samples were collected at 5 cm intervals. For grain size analysis, all samples weighing 3–5 g were boiled with 30% hydrogen peroxide (H_2_O_2_) to remove organic matter and 10% hydrochloric acid (HCl) to remove calcium carbonate, finally the remains were dispersed with 0.5 N sodium metaphosphotate ((NaPO_3_)_6_) solution and ultrasonicated for 10 min before measuring. Samples were measured using a Malvern Mastersizer 2000 laser grain-size analyzer at the Institute of Earth Environment, Chinese Academy of Sciences, which has a measurement range of 0.01~2000 μm with a 0.1 Φ interval resolution. Replicate analyses indicate that the mean grain size has an analytical error of <2%.

## Additional Information

**How to cite this article**: Li, Y. *et al*. Rapid and cyclic dust accumulation during MIS 2 in Central Asia inferred from loess OSL dating and grain-size analysis. *Sci. Rep.* **6**, 32365; doi: 10.1038/srep32365 (2016).

## Supplementary Material

Supplementary Information

## Figures and Tables

**Figure 1:**
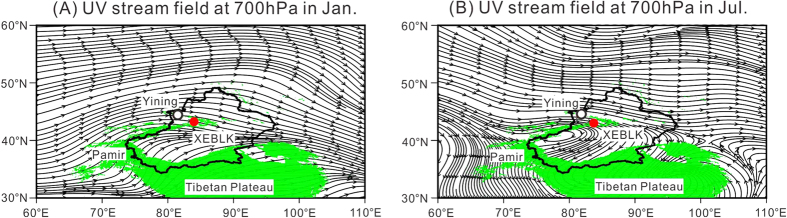
The UV stream field at 700 h Pa (about 3000 m a.s.l) in Xinjiang, west China based on the National Centers for Environmental Prediction/National Center for Atmospheric Research reanalysis: (**a**) January, and (**b**) July. The results indicate that the Ili Basin is controlled by the Westerlies all through the year. The UV stream field was gengerated using GrADS 2.0 software (http://grads.iges.org/grads/).

**Table 1:** Summary of dosimetry and OSL ages at XEBLK section.

Sample	Depth	U	Th	K	Water content	Dose rate	ED value	OSL age
No	(m)	(ppm)	(ppm)	(%)	(%)	(Gy/ka)	(Gy)	(ka)
XEBLK-0	0	2.966	12.92	2.197	15 ± 5	3.68 ± 0.31	1.68 ± 0.1	0.46 ± 0.05
XEBLK-2	2	4.984	12.33	1.803	15 ± 5	3.74 ± 0.31	37.67 ± 0.98	10.07 ± 0.87
XEBLK-4	4	2.876	10.83	1.723	15 ± 5	3.01 ± 0.26	35.6 ± 1.63	11.84 ± 1.16
XEBLK-6	6	3.373	11.19	1.981	15 ± 5	3.34 ± 0.29	50.8 ± 1.84	15.23 ± 1.4
XEBLK-8	8	3.009	11.92	1.745	15 ± 5	3.08 ± 0.29	53.43 ± 2.45	16.46 ± 1.65
XEBLK-10	10	3.583	10.92	1.564	15 ± 5	2.99 ± 0.26	52.27 ± 1.83	17.49 ± 1.65
XEBLK-12	12	3.372	10.7	2.057	15 ± 5	3.32 ± 0.3	61.86 ± 2.42	18.30 ± 1.81
XEBLK-14	14	3.745	13.1	1.993	15 ± 5	3.47 ± 0.3	67.96 ± 2.13	19.33 ± 1.77
XEBLK-16	16	3.06	11.72	2.066	15 ± 5	3.31 ± 0.31	67.0 ± 2.25	20.25 ± 1.99
XEBLK-18	18	3.506	12.64	1.543	15 ± 5	3.04 ± 0.26	63.9 ± 4.51	21.0 ± 2.34
XEBLK-20	20	3.285	9.809	1.698	15 ± 5	2.92 ± 0.26	59.58 ± 2.8	21.95 ± 2.34
XEBLK-22	22	2.797	11.24	1.689	15 ± 5	2.88 ± 0.27	66.1 ± 3.02	22.93 ± 2.4
XEBLK-24	24	2.894	11.13	1.944	15 ± 5	3.11 ± 0.29	68.57 ± 3.21	22.05 ± 2.28*
XEBLK-26	26	2.914	10.46	1.858	15 ± 5	2.95 ± 0.27	49.6 ± 0.8	16.8 ± 1.5*
XEBLK-28	28	2.991	11.67	1.793	15 ± 5	3.13 ± 0.29	81.0 ± 3.18	26.63 ± 2.70
XEBLK-30	30	3.256	12.07	1.479	15 ± 5	2.87 ± 0.26	83.4 ± 3.34	29.04 ± 2.85
