# Supplementary material for: Rapid and cyclic dust accumulation during MIS 2 in Central Asia inferred from loess OSL dating and grain-size analysis
Source: Sci Rep. 2016 Sep 2;6:32365. doi: 10.1038/srep32365 (PMC5009356; doi:10.1038/srep32365)
Supplement: Supplementary Information [file srep32365-s1.doc]

**Rapid and cyclic dust accumulation during MIS 2 in Central Asia inferred from loess OSL dating and grain-size analysis**

Yun Li^1,2^, Yougui Song^1^*, Zhongping Lai^3^*, Li Han^4^, Zhisheng An^1^

^1^State Key Laboratory of Loess and Quaternary Geology, Institute of Earth Environment, Chinese Academy of Sciences, 10 Fenghui Nan Road, Xi’an 710075, China.

^2^The Salt Lake Geology and Environment Laboratory, Qinghai Institute of Salt Lakes, Chinese Academy of Sciences, 18 Xining Road, Xinning 810008, China.

^3^ School of Earth Sciences, China University of Geosciences, Wuhan 430074, China.

^4^Salt Lake Analytical and Test Department, Qinghai Institute of Salt Lakes, Chinese Academy of Sciences, 18 Xining Road, Xinning 810008, China.

Table S1. Lithological description of the XEBLK loess section.

| Unit | Depth (m) | Details |
| --- | --- | --- |
| Surface soil | 0-0.7 | Light yellowish brown (10YR 6/4),fine to medium silt, massive and granular structure, numerous snails and fine roots |
| Loess | 0.7-9.0 | Light grayish yellow (2.5Y 6/3), medium to coarse silt, with little stratification, and are homogenous and massive with occasionally outcropped pellicle and fleck calcium carbonate |
| Weak paleosol | 9.0-12.0 | Light yellowish brown (10YR 6/4),fine to medium silt, massive and granular structure |
| Loess | 12.0-25.0 | light grayish yellow (2.5Y 6/3), medium to coarse silt, with little stratification, and are homogenous and massive with occasionally outcropped pellicle and fleck calcium carbonate |
| Paleosol | 25.0-30.7 | Yellowish red (5YR 5/6) to strong brown (7.5YR 5/6), fine silt, flecks and small (<0.5 cm)calcium carbonate nodules commonplace, manganese oxide staining on ped faces |


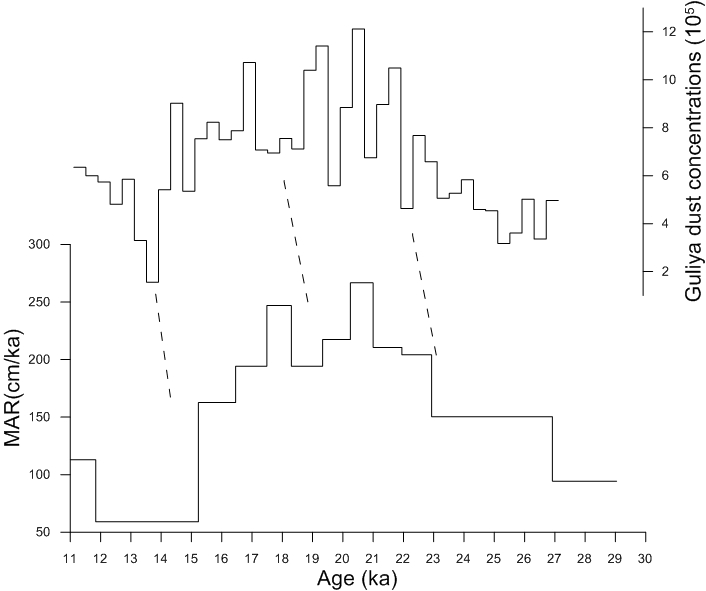


Figure S1. The MAR of XEBLK loess (bottom) and dust concentrations of Guliya[^1^](#_ENREF_1) ice core comparison during MIS2.


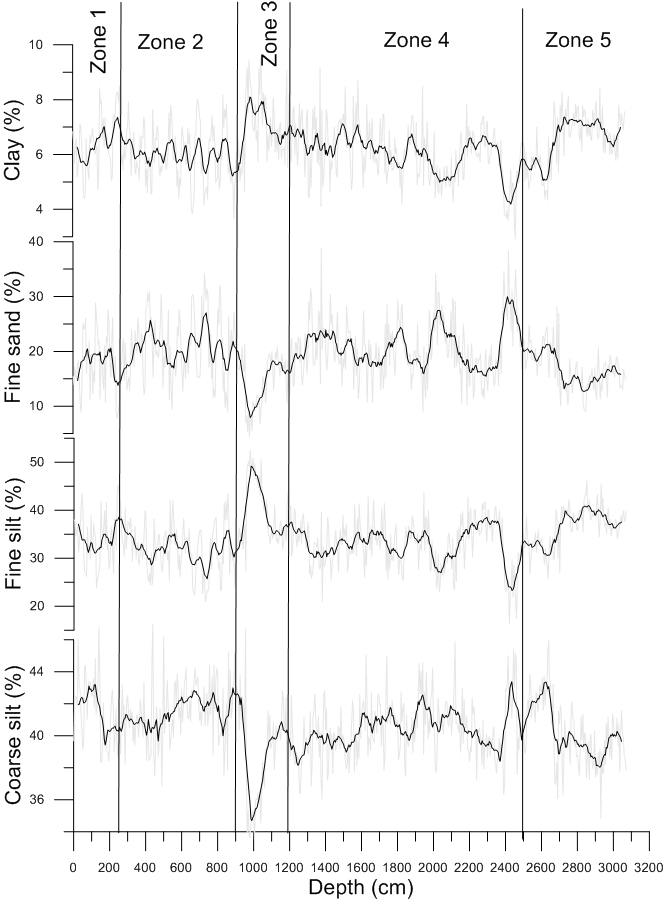


Figure S2. Grain size analysis of XEBLK loess (black, seven-point running mean).


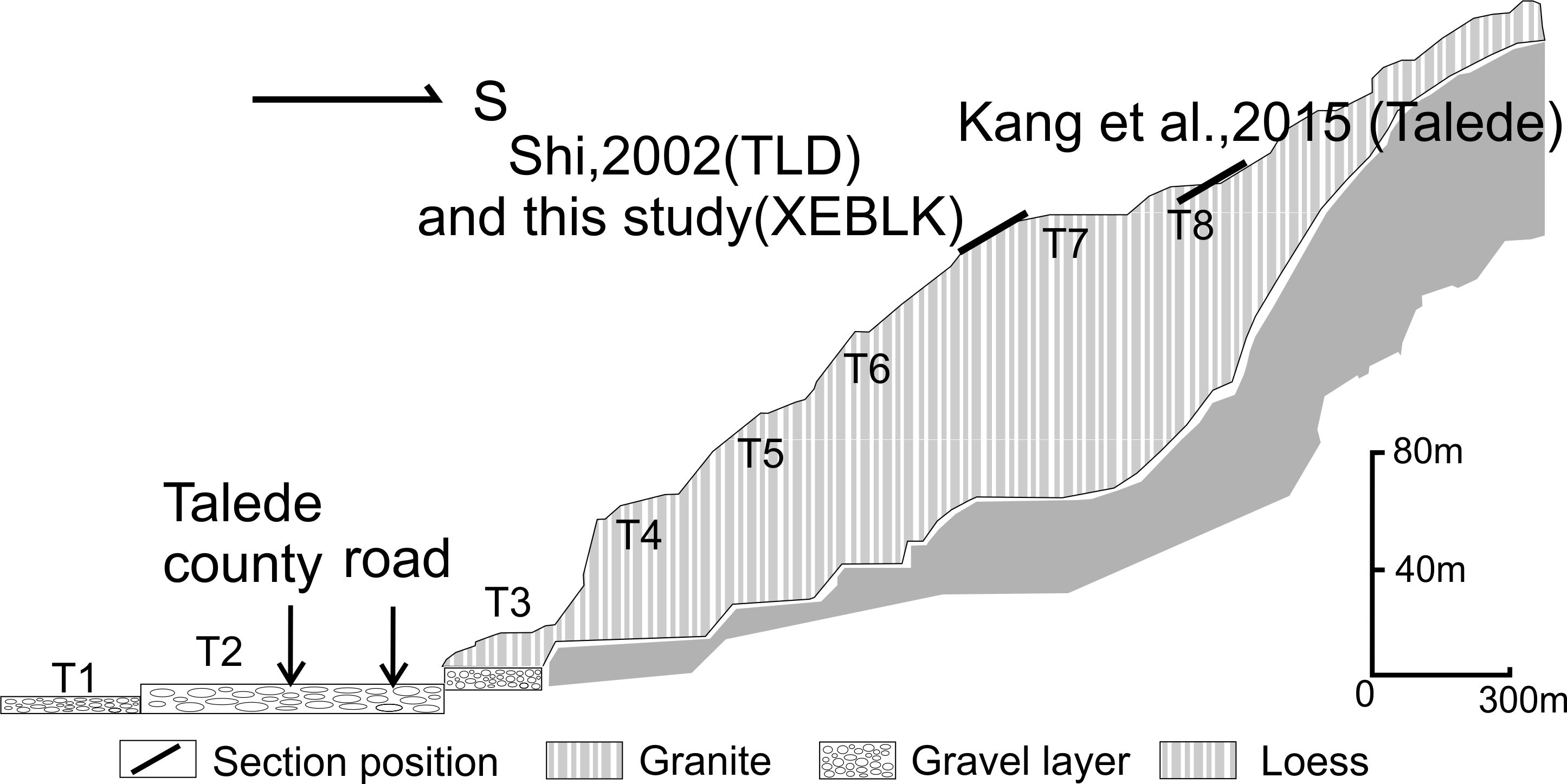


Figure S3. Location of our section, TLD[^2^](#_ENREF_2) and Talede section[^3^](#_ENREF_3) and terrace in southern side of Yili river.


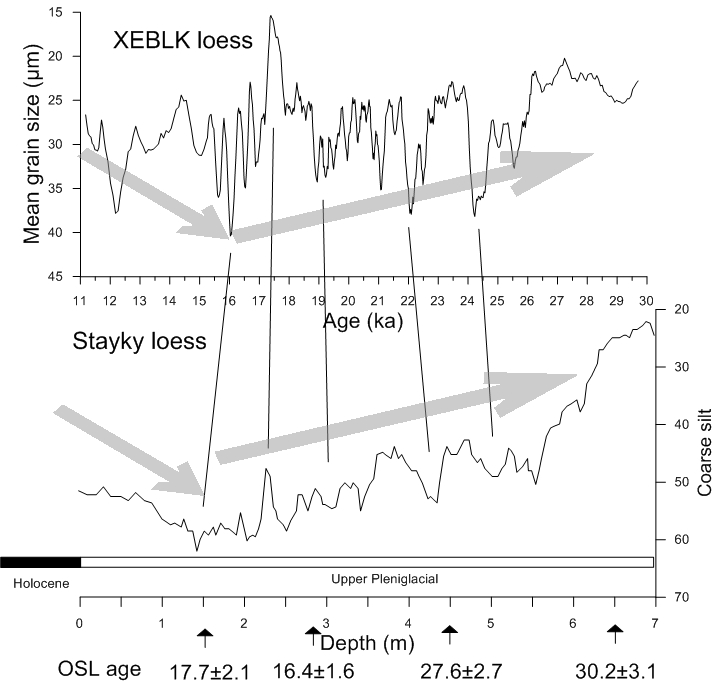


Figure S4. Grain-size comparasions of the XEBLK loess (this study) and Stayky loess[^4^](#_ENREF_4).


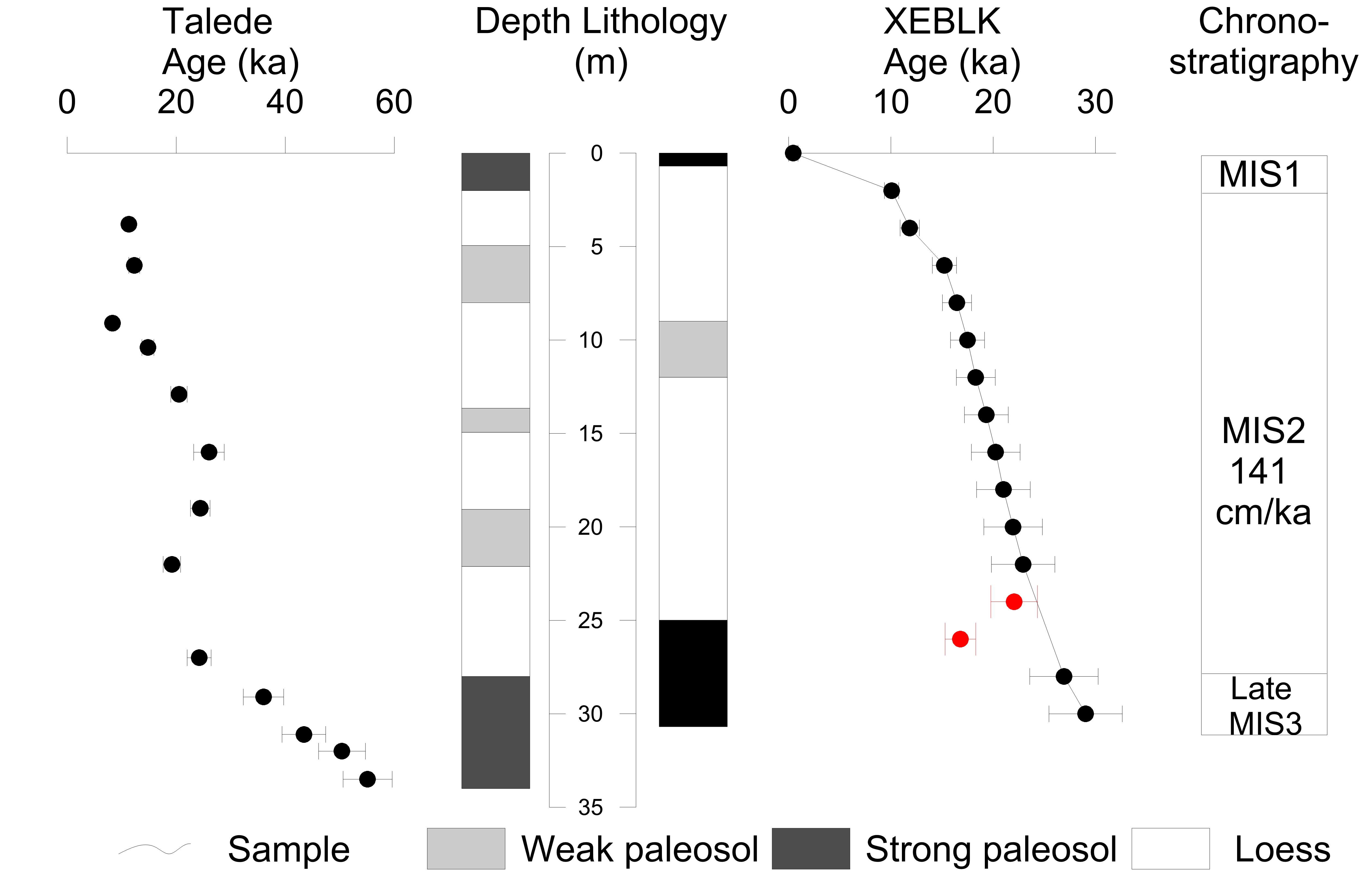


Figure S5. OSL ages and stratigraphy of XEBLK site and comparison with that of TLD site[^2^](#_ENREF_2).


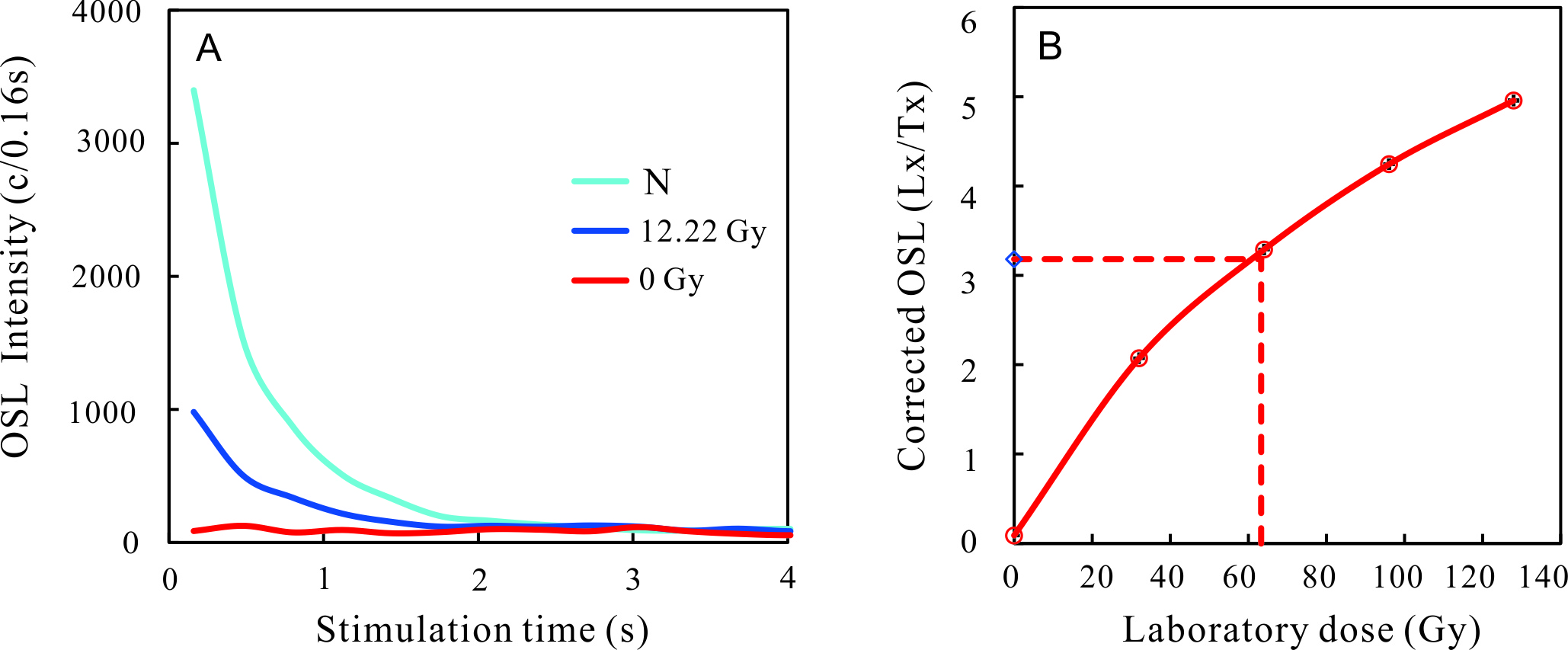


Figure S6. OSL decay curve of sample XEBLK-12 (A), and the dose response curve showing De determination.

**References**

1 Wu, G., Yao, T., Thompson, L. G. & Li, Z. Microparticle record in the Guliya ice core and its comparison with polar records since the last interglacial. *Chinese Science Bulletin* **49**, 607-611 (2004).

2 Shi, Z. *Loess in Tianshan Mountains and its implication of drying and desertification in Xinjiang, northwest China (in Chinese with English abstract).* Lanzhou university, (2002).

3 Kang, S. *et al.* A high-resolution quartz OSL chronology of the Talede loess over the past ∼30 ka and its implications for dust accumulation in the Ili Basin, Central Asia. *Quaternary Geochronology* **30, Part B**, 181-187 (2015).

4 Rousseau, D. D. *et al.* North Atlantic abrupt climatic events of the last glacial period recorded in Ukrainian loess deposits. *Clim. Past* **7**, 221-234 (2011).
